# Egg and Egg-Derived Foods: Effects on Human Health and Use as Functional Foods

**DOI:** 10.3390/nu7010706

**Published:** 2015-01-20

**Authors:** Jose M. Miranda, Xaquin Anton, Celia Redondo-Valbuena, Paula Roca-Saavedra, Jose A. Rodriguez, Alexandre Lamas, Carlos M. Franco, Alberto Cepeda

**Affiliations:** 1 Laboratorio de Higiene Inspección y Control de Alimentos, Dpto. de Química Analítica, Nutrición y Bromatología, Universidad de Santiago de Compostela, 27002 Lugo, Spain; E-Mails: pekenarv@gmail.com (C.R.-V.); procsaa@hotmail.es (P.R.-S.); alexandre.lamas@gmail.com (A.L.); carlos.franco@usc.es (C.M.F.); alberto.cepeda@usc.es (A.C.); 2 Clavo congelados, S. A. Caldas de Reis, 36650 Pontevedra, Spain; E-Mail: xaquin@clavo.net; 3 Centro de Investigaciones Químicas, Universidad Autónoma del Estado de Hidalgo, Carr. Pachuca-Tulancingo Km. 4.5, 42076 Pachuca, Hidalgo, Mexico; E-Mail: josear@uaeh.edu.mx

**Keywords:** egg, egg-derived products, functional foods, cholesterol, technological elaboration, omega-3.

## Abstract

Eggs are sources of protein, fats and micronutrients that play an important role in basic nutrition. However, eggs are traditionally associated with adverse factors in human health, mainly due to their cholesterol content. Nowadays, however, it is known that the response of cholesterol in human serum levels to dietary cholesterol consumption depends on several factors, such as ethnicity, genetic makeup, hormonal factors and the nutritional status of the consumer. Additionally, in recent decades, there has been an increasing demand for functional foods, which is expected to continue to increase in the future, owing to their capacity to decrease the risks of some diseases and socio-demographic factors such as the increase in life expectancy. This work offers a brief overview of the advantages and disadvantages of egg consumption and the potential market of functional eggs, and it explores the possibilities of the development of functional eggs by technological methods.

## 1. Introduction

Nowadays, foods are not intended to only satisfy hunger and to provide necessary nutrients for humans, but also to prevent nutrition-related diseases and improve physical and mental wellbeing of consumers [1]. However, human nutrition in developed countries is characterized by an excessive intake of protein, cholesterol, saturated fatty acids (SFA), *n-*6 polyunsaturated fatty acids (PUFA), calories or sodium, whereas consumption is deficient in *n-*3 PUFA, fiber and antioxidants. These imbalances are partly responsible for the high incidence of both obesity and the onset of chronic or degenerative non-transmissible diseases, from which cardiovascular diseases (CVD) are the leading cause of mortality and morbidity globally [2]. The consumption of a lower fat diet is generally accepted in all clinical guidelines on CVD prevention, and is based on total fat consumption of 25%–35% of total calories, of SFA should be no more than 7%–10%, *trans* fatty acids less than 1%, unsaturated fats, mainly monounsaturated fats (MUFA) and *n*-3 PUFA) should represent the rest of the calories from fat and cholesterol, for a total of less than 300 mg/day [3,4].

In order to improve public health, nutritional experts and related organizations, such as the U.S. Department of Agriculture and U.S. Department of Health and Human Services [5] of the Spanish Society of Community Nutrition (SENC) [6], has persistently recommended a reduction in the intake of foods that are related to the occurrence of chronic diseases, and am increased consumption of fruits and vegetables, grains, legumes, low-fat dairy products, lean meats and fish, especially fatty fish species that are high in *n-*3 PUFA. Owing to the persistence of these recommendations, there is a high degree of awareness of this problem in the populations of developed countries, and, fortunately, nutritional composition is already a major factor in the choice of foods by the consumer. However, although there is a significant demand for healthier food, consumers are reluctant to change their dietary habits [7]. This suggests that there is great potential for foods that are consumed regularly when they are converted to functional foods by changing the composition to include certain ingredients that are beneficial to health. Another way to obtain functional foods is to modify the quantity of certain components in the food to make it more suitable to the recommendations of nutrition experts [7].

In this sense, because eggs are a conventional food containing nutrients that play fundamental roles beyond basic nutrition, their promotion as functional foods should be considered [8]. Eggs are of particular interest from a functionality point of view, because they offer a moderate calorie source (about 150 kcal/100 g), a protein of excellent quality, great culinary versatility and low economic cost [9], which make eggs within reach to most of the population. Eggs are also relatively rich in fat-soluble compounds and can, therefore, be a nutritious inclusion in the diet for people of all ages and at different stages of life. In particular, eggs may play a particularly useful role in the diets of those at risk of low-nutrient intakes such as the elderly, pregnant women and children [10]. Additionally, it must be mentioned that eggs can be consumed throughout the world, having no use restrictions on religious grounds [11].

However, eggs are a controversial food for nutritional experts and health agencies, because of the saturated fat content (about 3 g/100 g) and cholesterol content (about 200–300 mg/100 g) [12]. Owing to these two characteristics, during the past 40 years, the public had been warned against frequent egg consumption due to the high cholesterol content in eggs and the potential association with CVD. This was based on the assumption that high dietary cholesterol consumption is associated with high blood cholesterol levels and CVD. Afterwards, subsequent research suggests that, in contrast to SFA and TFA, dietary cholesterol in general and cholesterol in eggs in particular have limited effects on the blood cholesterol level and on CVD [4].

However, the volume of eggs and egg yolk used by food companies in their formulations is constantly increasing. Nowadays, egg-yolk products are largely used by the food industry as a result of three very important properties: manufacture and stabilization of emulsions, foaming stability and thermal gelation, as it is a fundamental ingredient for the elaboration of several food products [13]. Unfortunately, eggs and egg-derived foods are responsible for a large number of food-borne illnesses each year, mainly caused by *Salmonella* [14]. For this reason, as well as for their lower price and ease of handling and storing compared to shelled eggs, the food service industry and commercial food manufacturers have shown an increasing interest in the use of liquid pasteurized egg products instead of fresh whole eggs [15].

Thus, it would be of major interest to develop egg-derived products, appropriate for food companies, with a modified nutritional composition that helps maintain the health of consumers. Nowadays, retail markets for functional eggs are available, mainly enriched with *n*-3 PUFAs or with low cholesterol content. However, in most cases, these eggs are obtained through modification of layer-hen’s diet and management, whereas much less attention has been paid to the development of functional eggs by means of technological methods [16]. In this manuscript, the possible development of functional pasteurized liquid eggs by technological methods and their advantages in the food industry and from the point of view of nutritionists are also discussed.

## 2. Advantages of Egg Consumption for Human Health

Eggs are an inexpensive and highly nutritious food, providing 18 vitamins and minerals, the composition of which can be affected by several factors such as hen diet, age, strain as well as environmental factors [16,17]. Nevertheless, although different compositions have been reported by several authors [10,17], on average, the macronutrient content of eggs include low carbohydrates and about 12 g per 100 g of protein and lipids, most of which are monounsaturated [8,18,19] and supply the diet with several essential nutrients (Table 1). Some of these nutrients, such as zinc, selenium, retinol and tocopherols, are deficient in people consuming a western diet, and given its antioxidant activity, can protect humans from many degenerative processes, including CVD [10].

There is also scientific evidence that eggs contain other biologically active compounds that may have a role in the therapy and prevention of chronic and infectious diseases. The presence of compounds with antimicrobial, immunomodulator, antioxidant, anti-cancer or anti-hypertensive properties have been reported in eggs [11]. Lysozime, ovomucoid, ovoinhibitor and cystatin are biologically active proteins in egg albumen, and their activity prolongs the shelf life of table eggs [14]. Some of these protective substances are isolated and produced on an industrial scale as lysozymes and avidin. Additionally, eggs are an important source of lecithin and are one of the few food sources that contain high concentrations of choline [8,20]. Lecithin, as a polyunsaturated phosphatidylcholine, is a functional and structural component of all biological membranes, which acts in the rate-limiting step of the activation of membrane enzymes such as superoxide dismutase. It has been suggested that ineffective activation of these antioxidant enzymes would lead to increased damage of membranes by reactive oxygen species. In addition, lecithin increases the secretion of bile, preventing stagnation in the bladder and, consequently, decreases the lithogenicity [8].

**Table 1:** Nutritional composition of hen eggs.

Component (Unit)	Amount	Component (Unit)	Amount
Egg shell (%)	10.5	Calcium (mg)	56.0
Egg yolk (%)	31	Magnesium (mg)	12.0
Egg white (%)	58.5	Iron (mg)	2.1
Water (g)	74.5	Phosphorus (μg)	180.0
Energy (Kcal)	162	Zinc (mg)	1.44
Protein (g)	12.1	Thiamine (mg)	0.09
Carbohydrates (g)	0.68	Riboflavin (mg)	0.3
Lipids (g)	12.1	Niacin (mg)	0.1
Saturated fatty acids (g)	3.3	Folic acid (μg)	65.0
Monounsaturated fatty acids (g)	4.9	Cyanocobalamin (μg)	66.0
Polyunsaturated fatty acids (g)	1.8	Pyridoxine (mg)	0.12
Cholesterol (mg)	410	Retinol equivalents (μg)	227.0
Iodine (μg)	12.7	Potassium (mg)	147
Tocopherols (μg)	1.93	Carotenoids (μg)	10
Selenium (μg)	10	Cholecalciferol (μg)	1.8

Quantities represent an edible portion of about 100 g.

However, as a component of egg lecithin, choline has numerous important physiologic functions, which include the synthesis of phospholipids, the metabolism of methyl and cholinergic neurotransmission, and it is a required nutrient that is essential for the normal development of the brain [21].

Another important nutritional component from eggs is phosvitin, a phosphoglycoprotein present in egg yolk and represents about 7% of yolk proteins. It has a specific amino-acid composition, comprised of 50% serine, and 90% of which are phosphorylated. This specific structure makes phosvitin a strong metal chelator and, by this mechanism, it acts as an important melanogenesis inhibitor to control excessive melanin synthesis in the melanocytes of animal and human skin [21]. It was suggested that egg-yolk phosvitin has the potential to be used as a natural bioactive compound as a hyper-pigmentation inhibitor for human skin [21].

Other interesting egg components from the nutritional point of view are the carotenoids. Carotenoids are natural pigments in hen egg yolks that confer its yellow color, which can range from very pale yellow to dark brilliant orange. Egg carotenoids represent less than 1% of yolk lipids, and are mainly composed of carotene and xanthophylls (lutein, cryptoxanthin and zeaxanthin) [19,22,23]. The total concentration of lutein and zeaxanthin is 10 times greater than of cryptoxanthin and carotene, combined [23], and are not endogenously synthesized by the human body and tissue levels therefore depend on dietary intake. These natural compounds found in the bodies of animals, and in dietary animal products, are ultimately derived from plant sources in the diet, mainly from dark green leafy plants [24]. Lutein and zeaxanthin content of eggs depends on different factors, such as the feed given to laying hens, or the husbandry system. Thus, variable contents of these carotenoids in non-enriched eggs were recently reported, varying about 167–216 μg/yolk for lutein and about 85–185 μg/yolk for zeaxanthin [22,24]. Additionally, a greater serum response to lutein was reported following the consumption of eggs compared with the consumption of dietary lutein supplements or vegetables [22,24]. This could be related with the fact that carotenoids depend on a lipophilic environment for optimal gastrointestinal uptake [24]. Consequently, eggs are a very important food source of these carotenoids, especially in the case of people that consume low amounts of vegetables with a high content of these substances (as occurs in western developed countries).

These carotenoids are, perhaps, best known for their function in the neural retina, where they are found in high concentration and, along with their isomer mesozaexanthin, are termed macular pigment [25]. Lutein and zeaxanthin are known to serve light-absorbing and blue-filtering optical functions, as well as antioxidant and anti-inflammatory functions, and thereby, is considered to play a role reducing immune-mediated macular degeneration and age-related cataract formation [23,24,25].

Taking into account the presence of all these components, eggs can be considered a nutritious inclusion in the diet for people of all ages and at different stages of life, but they may play a particularly useful role in the diets of those at risk of low-nutrient intakes [10]. Owing to their high nutritional value, eggs are also an important food that should be included in the planning of diets for patients, and are especially valuable in feeding people with gout, because it is a source of protein that does not add purines. Additionally, for people in sports training, egg proteins may have a profound effect on the training results, because, by its inclusion in the diet, it could be possible to enhance skeletal muscles synthesis [8]. It is well established that essential amino acids stimulate skeletal muscle protein synthesis in animal and human models, and the protein in egg has the highest biological value [26]. Fifteen grams of egg white protein contain about 1300 mg of leucine (the third most common amino acid in egg, after glutamic and aspartic acids), and is also an abundant source of branched amino acids and aromatic amino acids. Recent data showed that leucine induces a maximal skeletal muscle protein anabolic response in young people, which suggests that egg white protein intake might have an important effect on body mass accretion [27]. Specifically, leucine stimulates skeletal muscle synthesis independently of all other amino acids in animal models and is a potent stimulator of the cell hypertrophy mammalian target of rapamycin complex pathway. Additionally, leucine decreases muscle protein breakdown and breakdown-associated cellular signaling and mRNA expression [26].

## 3. Undesirable Effects of Egg Consumption

Despite their abovementioned nutritional benefits, egg consumption was traditionally associated with adverse factors for human health and nutrition. In this sense, egg whites contain anti-nutritional factors, among which are proteins such as ovomucoid that can inhibit trypsin or avidin, which can bind biotin. However, these factors are thermo-labile and, therefore, these compounds are usually destroyed when cooking eggs, after which they do not cause further detrimental effects. Additionally, eggs have been the subject of numerous recommendations from nutrition experts in order to moderate egg consumption, owing to its high cholesterol and saturated-fat content. Reducing saturated-fat intake is the primary dietary strategy recommended for reducing serum cholesterol levels, and this strategy has often led to a reduction in the consumption of eggs. Nevertheless, substituting eggs for other animal-protein foods in the diet may result in small changes to low-density lipoprotein cholesterol (LDL) [10] and, consequently, egg consumption should be considered in a similar way to other protein-rich foods. Although metabolic studies have shown that dietary cholesterol is a major determinant of serum cholesterol concentrations, other studies failed to detect changes in the serum total-cholesterol concentration when eggs were added to diets that already contained moderate amounts of cholesterol [28]. In this sense, large research works, and even meta-analyses, have been conducted to investigate the effects of eggs on serum cholesterol levels and cardiovascular health, with very different conclusions (Table 2). Several authors state that dietary cholesterol from eggs could be an important risk factor for cardiometabolic diseases including CVD and diabetes [12,29,30,31]. Furthermore, lecithin (approximately 250 mg in a large egg yolk) is converted by intestinal bacteria to trimethylamine, which is in turn oxidized by the liver to trimethylamine oxide, which is pro-atherosclerotic [32]. In this sense, a meta-analysis found that an intake increment of four eggs per week could possibly increase the risk of CVD by 6% and diabetes by 29% [12]. Nevertheless, a recent systematic review found no clear relation of egg consumption and CVD among diabetic individuals [33].

However, for a large number of researchers, traditional assumptions that dietary cholesterol consumption translates directly into elevated plasma cholesterol levels and the development of CVD in all individuals were deemed to be mistaken [10,34,35]. First, a conservative estimate suggests that only 30% of the population would hyper-respond to dietary cholesterol [29], whereas approximately 70% of humans are hypo-responsive to excess dietary cholesterol consumption [36]. Therefore, clinical studies have clearly shown that plasma compartment changes, resulting from dietary cholesterol consumption, are influenced by several factors such as ethnicity, genetic makeup, hormonal factors and body mass index [36,37]. All of these characteristics determine who would hyper-respond to dietary cholesterol and those who are hypo-responsive to intake. In addition, those individuals who hyper-respond to dietary cholesterol intake generally show increased LDL and high-density lipoprotein cholesterol (HDL) [8], allowing for the maintenance of the LDL/HDL ratio, an important marker for CVD risk. This suggests that, for healthy individuals, the nutritional benefits clearly outweigh the concern surrounding the dietary cholesterol provided by one large egg.

**Table 2:** Recent works regarding effect of eggs consumption on of serum cholesterol and cardio circulatory human health.

Reference	Study Design	Number and Type of Subjects	Main Conclusion
Djousse, Graziano, 2008a [30]	Prospective cohort study	21,275 US male physicians aged 40–85 years	Egg consumption of ≥ 1 per day was related to an increased risk of heart failure among male
Djousse, Graziano, 2008b [38]	Prospective cohort study	21,327 US male physicians aged 40–85 years	Infrequent egg consumption does not seem to influence the risk of CVD in male. In addition, egg consumption was positively related to mortality, more strongly so in diabetic subjects
Herron, Fernandez 2004 [8]	Expert Opinion	-	Current recommendation about limiting egg consumption are not benefiting the public as a whole and may have negative nutritional implications
Hu *et al.* 1999 [34]	Prospective study	37,851 men aged 40 to 75 years at study outset and 80,082 women aged 34 to 59 years at study outset, free of cardiovascular disease, diabetes, hypercholesterolemia, or cancer	Consumption of up to 1 egg per day is unlikely to have substantial overall impact on the risk of CHD or stroke among healthy men and women
Katz *et al.* 2005 [39]	Randomized crossover controlled trial	49 patients healthy adults with a mean age of 56 years	Short-term egg consumption does not adversely affect endothelial function in healthy adults
Li *et al.* 2013 [12]	Meta-analysis	320,778 included in 14 different studies	A dose-response association between egg consumption and the risk of CVD and diabetes
McNamara, 2000a [36]	Review	-	For the general population, dietary cholesterol makes no significant contribution to atherosclerosis and risk of cardiovascular disease
McNamara, 2000b [40]	Review	-	Cholesterol feeding studies demonstrate that dietary cholesterol increases both LDL and HDL cholesterol with little change in the LDL:HDL ratio
Nakamura *et al.* 2006 [28]	Prospective study	90,735 Japanese men and women aged 40–69 years	Eating eggs up to almost diary was not associated with an increase in CVD-incidence for middle-aged Japanese men and women
Nakamura *et al.* 2004 [41]	Prospective study	5186 Japanese women and 4077 Japanese men aged 40–69 years	Limiting egg consumption may have some health benefits, at least in women in geographic areas where egg consumption makes a relatively large contribution to total dietary cholesterol intake
Mutungui *et al.* 2008 [42]	Clinical trial	31 men aged 40–70 and with a Body Mass Index of 26–37	Including egg in a carbohydrate-restricted diet results in increasing HDL-C while decreasing the risk factors associated with metabolic syndrome
Natoli *et al.* 2007 [10]	Review	-	Egg consumption results in only a small increase in serum cholesterol levels in most people. The inclusion of eggs in the context of a diet low in saturated fat and containing cardio-protective foods is not associated with increased CVD risk.
Njike *et al.* 2010 [43]	Randomized placebo-controlled crossover trial	40 hyperlipidemic adults	Egg consumption was found to be non-detrimental to endothelial function and serum lipids in hyperlipidemic adults, while egg substitute consumption was beneficial
Quresci *et al.* 2007 [44]	Prospective study	9734 men and women aged 25 to 74 years	Consumption of greater than 6 eggs per week does not increase the risk of stroke and ischemic stroke
Rong *et al.* 2013 [35]	Meta-analysis	5847 incident cases for coronary heart disease, and 7579 incident cases for stroke	Consumption of up to one egg per day is not associated with increased risk of coronary heart disease or stroke
Scrafford *et al.* 2011 [45]	Prospective study	33,994 men and women free of CVD and completed food frequency questionnaire	It was not found a significant positive association between egg consumption and increased risk of mortality from CVD or stroke in the US population
Shin *et al.* 2013 [46]	Meta-analysis	A total of 16 studies were included, including participants ranging in number from 1600 to 90,735	Egg consumption is not associated with the risk of CVD and cardiac mortality in general population
Spence *et al.* 2012 [31]	Prospective study	1262 patients attending vascular prevention clinics, mean age of 61.5 years	Regular consumption of egg yolk should be avoided by persons at risk of cardiovascular disease
Spence *et al.* 2010 [47]	Review	-	It does exist evidence about people al CVD risk must to restrict egg consumption
Tran *et al.* 2014 [33]	Systematic Review	-	Confliting results prevent broad interpretation to conclude the effects of egg consumption and cardiovascular disease among diabetic individuals
Voutilainen *et al.* 2013 [48]	Prospective study	2682 middle-aged men	No evidence was found that people with cardiovascular risk should restrict egg consumption
Weggemans *et al.* 2001 [29]	Meta-analysis	17 studies including 556 subjects, 422 men and 134 women	Dietary cholesterol from eggs raises the ratio of total to HDL cholesterol and, therefore, adversely affects the cholesterol profile
Zampelas 2012 [49]	Invited Commentary	-	Although it becomes increasingly clearer that, eggs consumption is not associated with CVD risk in healthy populations, the evidence cannot be considered as conclusive in high risk populations
Zazpe *et al.* 2011 [50]	Prospective study	14,145 Mediterranean university graduates	No association between egg consumption and the incidence of CVD was found

In addition to the consumer individual response, there are other important factors of egg cholesterol that can play an important role in the effect on human health, such as the food matrix in which it is presented or the total diet consumed. Thus, previous studies have suggested that egg-yolk consumption raises serum cholesterol to a greater extent than crystalline cholesterol dissolved into a solution or incorporated into a diet [51]. On the other hand, another important factor in the individual response to egg cholesterol is the diet consumed. The egg consumption in countries with typical western diets, such as the United States, accounts for 26%–32% of the total dietary cholesterol intake, whereas egg consumption accounts for about 48% of total dietary cholesterol intake in Japan [28]. These differences are of major interest from a nutritional point of view, because an increase in serum cholesterol in response to increased egg consumption is 1.7 times greater when the background diet is high in saturated fat compared with a low-saturated-fat background diet. The effect may be attenuated even further in the case of overweight, insulin-resistant people [10].

In addition to nutrition-related risks, egg consumption can also represent a risk for consumers derived by other factors, such as their microbiological status. *Salmonella*, and serovars Enteritidis and Typhimurium are responsible for most food-borne illnesses associated with the consumption of eggs and egg products. In Europe, S. Enteritidis and S. Typhimurium are the most commonly isolated serotypes in human cases of salmonellosis, and contaminated eggs still remain the most important source of infection with S. Enteritidis for humans [14].

In fact, between 1993 and 2002, 9364 food-borne outbreaks were reported in Spain, 4944 (52%) of which were caused by *Salmonella* and 3546 (37.8%) of which were associated with egg products, constituting an obstacle to the well-being of populations and a source of high economic loss [52].

In order to have safe products, European Commission [53] requires the absence of *Salmonella* in 25 g or 25 mL of eggs and egg-derived foods, and limits the presence of Enterobacteriaceae to a maximum of 100 cfu/g after the pasteurization treatment. Limits are also given for 3OH-butyric acid (10 mg/kg dry matter), an index of the presence of incubator reject eggs, and for lactic acid (1000 mg/kg dry matter), a chemical index of hygienic quality of the raw material, which are to be measured before treatment [54]. Additionally, to ensure their safety, egg shells must be clean, dry, fully developed, and with no cracks; although, cracked eggs can be used if they are processed as soon as possible [55]. Eggs must be broken in a manner that minimizes contamination, from the shells themselves in particular, and egg contents may not be obtained by centrifuging or crushing the eggs [54].

Despite this strict safety normative, in some countries, the use of fresh eggs to elaborate egg-derived products in restoration is not allowed. For this purpose, it is mandatory to use pasteurized egg products. Although food-service industries other than central kitchens, caterers and restaurants are not bound to use pasteurized egg products, they have shown an increasing interest in their use, because of its convenience and ease of handling and storing compared to shelled eggs [15].

Another important human-health risk related to egg consumption is the potential presence of residues of veterinary drugs, because laying hens treated with pharmaceutical products can produce contaminated eggs [56]. Certain habits can also compromise health by being a source of exposure to environmental contaminants. Many of these potentially toxic pollutants are fat soluble, and thus, any fatty foods (including eggs) may often contain high levels of persistent organic pollutants [57] or dioxins, that are usually present even in free-range and organic eggs [58].

Additionally, egg allergies represent one of the most common IgE-mediated food allergies in infants and young children [59]. This allergy can be influenced on several environmental or demographic factors. Thus, a recent study found that factors as female gender, preterm delivery, having older siblings, maternal smoking during pregnancy or exposure in the first year to pets inversely associated with egg allergy. With respect to demographic origin, this work found that child with a family history of allergy and those parents born in East Asia are at increased risk of egg allergy [59]. Among infants and young children, egg allergy is the second most common food allergy after cow’s milk allergy [60]. The overall prevalence of egg allergy in children in the Western Countries is about 1%–3% [61], with prevalence in European countries about 2.5% [60,62].

The five major allergens identified in hens eggs are ovomucoid (Gal d1), ovalbumin (Gal d2), ovotransferrin (Gal d3), lysozyme (Gal d4) and albumin (Gal d5). The majority of allergenic proteins are contained in egg white (Gal d1–4) rather than in egg yolk (Gal d5) [63]. Several other allergens have been identified in egg yolk, including vitellenin (apovitellenin I) and apoprotein B (apovitellenin VI), although their role remains jet unclear [64]. However, various studies have demonstrated that a large number of egg allergic people were able to tolerate heated egg whites [65], an advantage for thermally processed eggs. Recent publications indicate that up to 70% of children with egg allergy can tolerate egg baked in a cake or muffin without apparent reaction. Heat treatment destroys the conformational epitopes that some individuals form IgE against, thus allowing ingestion of the egg without any adverse reaction. In addition to altering the epitopes, heating the egg protein also acting to reduce the allergenicity of the protein by affecting the digestibility of the proteins or making the IgE binding sites less accessible [66].

## 4. Recommendations and Worldwide Consumption of Eggs

Guidelines from agencies as the U.S. Department of Agriculture and U.S. Department of Health and Human Services [5] or the SENC [6] advise healthy adults to limit dietary cholesterol intake to less than 300 mg each day. However, due to the growing body of scientific literature showing a lack of relationship between egg intake and CVD, recent dietary guidelines indicate healthy people can consume one egg a day as part of a healthy diet [33]. Other guidelines have yielded different points, ranging from no restriction to recommending regular intake of eggs [67]. Despite the recommendations to limit egg consumption, the worldwide production of eggs increased in recent decades and exceeded 64 million tons in 2009, with China as the largest world producer, with 36% of the world’s production. For consumers, Mexico is the highest consumer per capita, reaching an average consumption of 355 eggs per person and year, followed by China (344) and Japan (325) [68]. The increase in worldwide egg production and consumption is rational, because egg protein is of excellent quality and low economic cost, whereas a big demand for protein sources are needed in developing countries, in which a third of the population are under nourished [69]. Additionally, the fact that eggs are a good food alternative for the elderly plays an important role in their consumption increase, because it is expected that, by the year 2020, the number of people worldwide over the age of 60 could reach one billion [8]. Although elevated total seric cholesterol values have been shown to predict CVD in middle-aged individuals, this parameter does not seem to be relevant for the elderly demographic. Unfortunately, in the elderly, the restriction of fat and cholesterol from the diet often results in the subsequent inclusion of foods that are high in simple sugars. This change in diet composition can be detrimental, causing increases in seric triglycerides (TG), which are generally accompanied by low HDL levels, which has been identified as the best lipoprotein indicator of CVD risk in elderly individuals. Furthermore, it has been suggested that the consumption of a low-fat diet by elderly individuals may promote insulin resistance as a consequence of increased levels of LDL and TG and decreased HDL levels in serum [8].

Furthermore, another important factor that could raise egg consumption in the near future is the fact that typical factors of modern life, such as frequent travel, busy schedules, little time to cook and eat at home and the inability to eat together as a family, play an important role in the increased consumption of pre-cooked and processed foods. As eggs are common ingredients employed by the food industry for their thickening, gelling, emulsifying, foaming, coloring, and flavoring properties, it is also expected that the worldwide consumption of eggs included in food industry formulations will increase in next years [13].

However, in the case of pre-cooked and processed foods, the use of pasteurized liquid eggs and egg powders are more commonly used than fresh eggs [70]. Food industries chiefly make use of the liquid egg products obtained through the shelling and pasteurization of shelled eggs, and whole egg products are employed as ingredients for the manufacture of egg pasta, mayonnaise, pastry or baked foods [13]. The pasteurization process can accelerate reactions between lipids and molecular oxygen, resulting in losses of nutritional and sensory properties of the egg products. Besides the possible impact of processing on lipid oxidation, the initial composition of raw materials can impact the behavior during processing [70].

Thus, there is a large potential market for functional egg products fortified with bioactive compounds by means of technological methods. Fortification is often the more cost effective and practical way to provide micronutrients to communities in need, especially if the technology already exists and if an appropriate and equitable food-distribution system is in place. It is usually possible to add multiple micronutrients without substantially increasing the total cost of the food product at the point of manufacture [69], especially when they manufacture large quantities of foods.

## 5. Potential Markets for Egg-Derived Functional Foods

The increasing demand for functional foods during recent decades can be explained by the increasing cost of healthcare, the steady increase in life expectancy and the desire for an improved quality of life in later years. Functional foods may improve the general condition of the body, decrease the risk of some diseases and may even be used to cure some illnesses. Taking into account the progressive aging of the population of developed countries, functional foods are a good alternative for controlling health costs, because medical services for the aging population are rather expensive [1].

Although the term “functional food” has already been defined several times, there is no unitarily accepted definition for this group of foods. In most countries, there is no legislative definition for the term and drawing a line between conventional and functional foods is challenging, even for nutritionist or food experts. The European Commission’s Concerted Action on Functional-Food Science in Europe (FuFoSE), coordinated by International Life Science Institute (ILSI) Europe stated that “a food product can only be considered functional if, together with the basic nutritional impact, it has beneficial effects on one or more functions of the human organism, thus either improving the general and physical conditions or/and decreasing the risk of the evolution of diseases. The amount of intake and form of the functional food should be as it is normally expected for dietary purposes. Therefore, it could not be in the form of a pill or capsule, just as normal food form” [1].

Experts like Sloan [71] has estimated the global functional-food market to be 47.6 billion US$, with the United States as the largest market segment followed by Europe and Japan. Some estimations report an even higher global market value (nearly 61 billion US$) [72]. The three dominant markets (USA, EU and Japan) contribute to over 90% of total sales, from which the European market was estimated to contribute around 15 billion US$ in 2006 [73]. It should be emphasized, however, that the European market is heterogeneous, and there are large regional differences in both the use and acceptance of functional foods. In general, the interest of consumers in functional foods in the Central and Northern European countries is higher than in Mediterranean countries, where consumers have appreciated natural, fresh foods and consider them better for health [1]. Additionally, women tend to be slightly more health-oriented than men, and middle-aged and elderly consumers tend to be substantially more health-oriented than younger consumers [74]. It has been suggested that the reason behind women’s higher awareness of health issues is the heightened responsibility they feel for the wellbeing of other family members (related to the dominant role of women as the main purchasers of foods in households). Therefore, middle-aged and elderly consumers are more aware of health issues, simply because they, or members of their immediate social environment, are much more likely to be diagnosed with a lifestyle-related disease than younger consumers. However, other recent research based on surveys did not find clear differences in the acceptance of functional foods between ages, gender or the country of origin of consumers [75].

It can be assumed that functional foods represent a sustainable category in the food market [76,77], because it cannot be neglected that functional-food products help to ensure an overall good health and/or to prevent/manage specific conditions in a convenient way [71,72]. Moreover, it is beyond doubt that persuading people to make healthier food choices would provide substantial health effects; therefore, it is in common economic and public interest [77]. This increasing consumer awareness, in combination with advances in various scientific domains, provides companies with unique opportunities to develop a large variety of new functional-food concepts [76]. It should also be considered that functional foods are sold at higher prices, thus containing larger profit margins than conventional foods, which obviously make the sector attractive for the players in the supply chain [73]. In general, price premiums of 30%–50% are observed in high-volume functional-food segments; however, for some products, it can be increased up to 500% [72,73].

Taking into account that eggs and egg-derived products are largely accepted by consumers, owing to their great culinary versatility and low economic cost, the development of functional eggs and egg-derived products could be an important way to value the products and to gain profitability for egg producers and the food industry [8]. Nevertheless, as previously reported [75], functional eggs are still rarely consumed in Europe. Recent polls revealed that consumers mentioned the consumption of functional eggs in only two countries. In Sweden, only 3.8% of those asked consumed eggs enriched with *n*-3 PUFAs, whereas in Spain, only 6.7% of the respondents consumed eggs enriched with *n*-3 PUFAs or eggs that were low in cholesterol. Consumers in all other surveyed European countries reported using no eggs or functional egg products [75].

## 6. Egg-Derived Products with High Omega-3 Fatty Acid Content

Although the possibility of using other bioactive compounds to obtain functional eggs, such as lycopene [78], was investigated, the most common bioactive compounds used for this purpose were *n*-3 PUFAs [16]. These fatty acids, especially eicosapentaenoic acid (EPA) and docosahesaenoic acid (DHA), have received great attention from nutritionists and the medical community, because it is considered that a clear relationship exists between the consumption of EPA and DHA and the maintenance of normal cardiac function. Thus, *n*-3 PUFA-fortified products (such as eggs) provide a means to achieve desired biochemical effects of these nutrients, without the ingestion of dietary supplements, medications or the need for a major change in dietary habits [19].

Most international agencies and sanitary authorities of western countries recommend a daily intake of *n*-3 PUFA between 1000 and 2000 mg daily [19], almost 200 mg/day of which should come from DHA. Owing to differences in the biological effectiveness, about ten times as much alpha-linolenic acid (ALA) is required to achieve a similar benefit to EPA and DHA. For this reason, European Commission [79] states that, in order to advertise and label food as “source of omega-3 fatty acids”, a food must contain at least 0.3 g of ALA per 100 g and per 100 kcal, or at least 40 mg of EPA + DHA per 100 g and 100 kcal, whereas in order to advertise and label food as “high omega-3 fatty acids’, it must contain at least 0.6 g of ALA per 100 g and 100 kcal, or at least 80 mg of EPA + DHA per 100 g and 100 kcal.

The content of *n*-3 fatty acids in eggs and egg-derived products can be increased, either through feed modifications for hens or through technological methods (in the case of egg-derived products). Depending on whether or not we want to produce a product that gets the statement “source of omega-3 fatty acids” or “high in omega-3”, we may choose to supplement the product with a specific raw material. Thus, if we want to increase the content of ALA, we can use plant oils as a source. Various plants, such as canola, soybean, walnuts and flaxseed, produce ALA, with the latter being the most concentrated source [80]. Consequently, flaxseed is the most employed matter for the supplementation of hens when aiming to obtain *n-*3 PUFA-enriched eggs by means of increasing the ALA content [16]. However, one of the disadvantages of flaxseed supplementation is that, when the hens feed under such production parameters, the egg characteristics are very contradictory in terms of feed consumption, egg production or egg weight [16].

On the contrary, owing to their EPA and DHA content, marine products, such as fish oils, seaweed or microalgae could be used to obtain egg-derived products with “sources of” or “high in” *n*-3 PUFA [16]. When using fish oil as source of *n*-3 PUFA, it is highly recommended to include an antioxidant substance to prevent sensorial hurdles that are mainly caused by oxidized *n*-3 PUFA in eggs [16]. In this sense, recent research has shown that, when seaweed is used as source of *n-*3 PUFA, it can also act as an antioxidant, as seaweed naturally contains antioxidants such as carotenoids, polyphenols, and vitamins E and C [81]. Additionally, inclusion of fish oil in the hens’ diets at levels above 1.5% generates eggs that are generally unacceptable to western consumers, with tastes described as “fishy”. In this sense, previous works observed that deodorization of fish oil, in order to reduce the amount of volatiles in the hens’ diet, did not improve the acceptability of the eggs [82]. Similarly, feed supplementation with microencapsulated fish oil, which is expected to have greater oxidative stability, still had a negative impact on egg sensory attributes [83]. Oxidative damage in egg yolks fat results only from direct deposition of oxidized lipids from the feed, as lipids are not further oxidized during storage [16,80].

With respect to the microalgae supplementation of hens’ diets, autotrophic or heterotrophic microalgae can be employed. For autotrophic microalgae, despite being an excellent source of *n-*3 PUFA and other important bioactive compounds such as carotenoids [84], the high price of production restricts its application in relatively low-value products such as eggs [16]. With respect to heterotrophic microalgae, recent technology has been developed to produce marine microalgae with an extremely high DHA content (about 18% of dry mass) through a fermentation process. Oils obtained of two microalgae, sources of *n-*3 PUFA, has yet obtained authorization by the European Commission to be employed as novel food ingredients, (*Schizochytrium* sp. and *Ulkemia* sp.) [85,86]. Eggs from hens fed heterotrophic microalgae typically show similar PUFA profiles to eggs from hens fed fish oil, yielding eggs with DHA contents up to 200 mg per egg, while maintaining consumer acceptability [16].

Given the relatively limited conversion of ALA into EPA and DHA by the human metabolism, feed supplementation with long chain *n*-3 PUFA in the form of fish oil or microalgae is much more interesting compared to supplementation with their precursor ALA through the addition of flaxseed. In any case, the obtained enriched eggs by supplementation of hens diet does not affect the cholesterol content of the eggs [87] and, consequently, consumers could be reluctant to consume eggs as a source of *n*-3 PUFA, owing to their high cholesterol content [29,34].

Therefore, one way to diversify the supply and to possibly enlarge the market of egg products is to produce *n*-3 PUFA-enriched pasteurized liquid eggs and egg powders. Using technological methods, it is possible to fortify these by-products with *n*-3 PUFAs at the same time as reducing cholesterol content. Moreover, their use as ingredients in a wide range of processed foods could contribute to increased consumption of *n*-3 PUFA among the population [70]. Another important potential advantage of egg-derived products enriched with *n-*3 PUFA is that the lipid profile is better preserved at refrigeration temperatures, because storage at room temperature results in a loss of PUFAs [9]. In some countries, such as those in the EU, it is established that fresh eggs must be stored and transported at a constant temperature and should, in general, not be refrigerated before sale to the final consumer (with the exception of French overseas departments) [88]. However, some egg-derived products, such as pasteurized liquid eggs, are required to be conserved by refrigeration. Thus, in the case of bioactive compounds that need refrigeration for better conservation, this could be an advantage for functional egg-derived products obtained by technological methods with respect to functional fresh eggs obtained by hens’ diet supplementation.

## 7. Eggs and Egg-Based Products with Low Cholesterol and Saturated Fats Contents

Eggs represent the major excretory route of the sterol in hens [89], which is almost entirely contained in the yolk. Different strategies were employed in order to obtain eggs with lower amounts in cholesterol. Most of these strategies have focused on genetic selection or alteration of the laying hens’ diet, with various nutrients, non-nutritive factors and pharmacological agents [89,90,91]. Unfortunately, the vast majority of these experimental approaches only elicited minimal changes (<10%), at best, or, as in the cases of some strategies such as dietary azasterols and triparanol, they resulted in the unacceptable replacement of yolk cholesterol by desmosterol [91]. The relatively poor effectiveness of strategies carried out for reducing yolk cholesterol content was probably due to the relative resistance of yolk cholesterol content to manipulation by genetic selection. Additionally, there is a lack of available pharmacological agents that could greatly attenuate hepatic cholesterol biosynthesis and metabolism, and/or lipoprotein assembly and secretion, without causing a cessation of egg production [89]. In this sense, copper supplementation to laying hens at pharmacological concentrations (>250 mg/kg) has been demonstrated to cause a reduction in egg-yolk cholesterol [92], although other researchers did not find differences in egg-yolk cholesterol after feeding pharmacological levels of dietary cooper [93,94]. On the other hand, the use of atorvastatin in laying hens elicited favorable changes in egg nutrient composition in addition to the reduction in egg-yolk cholesterol. Thus, eggs obtained from hens treated with atorvastatin were lower in fat and higher in high-quality proteins that control the eggs [90]. Regarding modification of hens’ diets, feeding hens a diet containing high amounts of fats or oils generally elevates the yolk cholesterol content [89], whereas feeding hens a diet containing low amounts of fats or oils slightly reduces the yolk cholesterol content. Other effective approaches to egg cholesterol reduction include feeding hens garlic paste [95].

In addition to strategies based hen genetic selection or on the modification of the feeding conditions of laying hens, it is also possible to produce eggs that are low in cholesterol by technological methods (Table 3). The simplest way is implemented after dehydrating the yolks and whites separately, developing a new “mix” with more content and less clear yolk, which is where the cholesterol fraction is found [96]. Another strategy is the removal of cholesterol from egg yolks using organic acids such as acetone [97]. However, the use of organic acid reduces the emulsifying capacity of the egg yolk, so detracts technological potential as an ingredient, and could potentially leave residues of these acids in the egg. Another option is the use of supercritical CO_2_, an extremely potent solvent for removing cholesterol from egg yolk [98]. This methodology has obtained very promising results, but despite its potential, it is not used a lot on the industrial level because of its high price. Other recent methodology described for this purpose includes the selective degradation of cholesterol in egg yolks using the enzyme cholesterol oxidase together with ultrasound [99] or using different species of microorganisms [100,101,102,103]. Alternatively, cholesterol can be removed from egg yolks using microbial degradation or by complexation with β-cyclodextrin, alone or inmobilized in chitosan beads [104,105], high metoxyl pectins [106], Streamline Phenyl^®^ resin [107] anionic chelating agent [108] or low-cholesterol liquid food oil [109,110], or by obtaining egg yolk granules [111].

**Table 3:** Different strategies employed to obtain egg-derived foods with lower amounts in cholesterol by technological methods.

Reference	Method Employed	Results Obtained
Aihara *et al.* 1998 [100]	Degradation of egg yolk cholesterol by *Rodococcus equi* No. 23	Degradation of about 60% of egg yolk cholesterol content
Chiu *et al.* 2004 [105]	Cholesterol absorption by β-cyclodextrin inmobilized in chitosan beads	Reduction of 92% of cholesterol content in egg yolk
Christodoulou *et al.* 1994 [101]	Bioconversion of cholesterol by 3 days of incubation with cholesterol oxidase from *Pseudomonas fluorescens* and *Nocardia erythropolis*	93% of egg yolk cholesterol bioconversion
Froning *et al.* 2008 [98]	Extraction of cholesterol from dried egg yolk using supercritical carbon dioxide	Reduction of about 2/3 of total cholesterol content in egg yolk
Garcia-Rojas *et al.* 2007 [106]	Extraction of cholesterol in liquid egg yolk using high methoxyl pectins	The egg yolk contends of cholesterol decreased about 14%
Garcia-Rojas *et al.* 2006 [107]	Removing egg yolk plasma cholesterol using Streamline Phenyl^®^ resin	70% of egg cholesterol content decreased in yolk plasma
Hsieh *et al.* 1994 [108]	Removing cholesterol and fat from egg by an anionic chelating agent (gum arabic)	Obtaining egg yolk essentially free from cholesterol
Jackeschky 2001 [109]	Dehydrating whole eggs or egg yolks and there upon treating it with an extraction based on a low-cholesterol, liquid food oil	Cholesterol proportion in the egg yolk being lowered by at least 95%
Kijowski, Lombardo 2000 [110]	Removing cholesterol from egg yolk by shearing a mixture of oil:egg yolk:water ratio	Reduction in cholesterol content about 50%–82% in egg yolk
Laca *et al.* 2014 [111]	Different methods to obtain egg yolk granules	Reduction in cholesterol content about 80%–90% in egg yolk granules with respect to egg yolk
Lv *et al.* 2002 [102]	Bioconversion of yolk powder cholesterol by extracellular cholesterol oxidase obtained from a mutant *Brevibacterium* sp	More than 85% of the yolk powder cholesterol was bioconverted
Manohar *et al.* 1998 [104]	Extraction of cholesterol from egg materials based on the use of β-cyclodextrin	Reduction of about 94.5% of total egg yolk cholesterol and esters
Martucci *et al.* 1997 [97]	Extraction of cholesterol from dehydrated hen egg yolk using acetone as solvent	Reduction about 91% of cholesterol content in egg
Sotelo, González 2000 [96]	Elaboration o fan egg powder mixture with a 3:1 proportion of white and yolk	Reduction about 40% of cholesterol content in egg
Sun *et al.* 2011 [99]	Ultrasonic-assisted enzymatic degradation	Cholesterol level in egg yolk was reduced to 8.32% of its original concentration without affecting the quality attributes of the yolk
Valcarce *et al.* 2002 [103]	Biocenversion of egg cholesterol to into pro-vitamin D sterols by the non-pathogenic ciliate *Tetrahymena thermophila*	Cholesterol content in egg yolk was reduced in about 55%

## 8. Conclusions

Eggs represent a very important food source, especially for some populations such as the elderly, pregnant women, children, convalescents and people who are sports training. The volume of both fresh eggs and eggs used by food companies in their formulations increases constantly. Owing to their higher security, lower price and easier handing and storing properties, food manufacturers prefer to use pasteurized egg products rather than fresh eggs. Additionally, the number of functional-food markets has also increased in recent decades and, owing to some factors such as the progressive aging of the population of developing countries, are expected to continue to increase in the coming years. Nevertheless, the presence of functional eggs in the market and knowledge of such products by the consumers are lower than other groups of foods.

Consequently, the development of functional egg-derived foods through technological methods could be an interesting way to gain profitability for egg producers and the food industry, in addition to improving the general conditions of public health. This could be especially interesting for the addition of bioactive compounds that need to be stored at refrigeration temperatures, because egg-derived products such as pasteurized liquid eggs must be stored under refrigeration during the commercialization process. Additionally, these products are safer from the microbiological point of view, cheaper, easier to hand and store, and because of the heat treatment applied, in some cases are less allergenic than fresh eggs. Thus, functional egg-derived products obtained through technological methods are a very interesting option for food manufacturers.
